# Cognitive Load Across Interaction Formats in Digital Attention Assessment for Children: Within-Subject Neuroimaging and Behavioral Comparison Study

**DOI:** 10.2196/93468

**Published:** 2026-05-26

**Authors:** Harim Jeong, Yong Jeon Cheong, Jihyeong Ro, Jihyun Cha, Jongkwan Choi, Minyoung Jung

**Affiliations:** 1 Korea Brain Research Institute Dong-gu, Daegu Republic of Korea; 2 OBELAB Inc Seoul Republic of Korea

**Keywords:** child, children, cognitive load, digital health, functional near-infrared spectroscopy, fNIRS, human-computer interaction, Stroop test

## Abstract

**Background:**

Digital health technologies increasingly use tablet-based cognitive assessments for children, yet interaction design choices can substantially influence cognitive load and measurement validity. Although cognitive load has been extensively studied in educational settings, its impact on patient-facing digital assessment tools for pediatric populations remains underexplored.

**Objective:**

This study examined how the interaction format influences cognitive load and measurement validity in a tablet-based Stroop task for children, comparing text-based with color-based response selection to determine which format better supports valid attention assessment.

**Methods:**

Using a within-subject design, 127 typically developing children (n=55, 43.3%, girls; n=72, 56.7%, boys) aged 6-12 (mean 9.15, SD 1.56) years were recruited via convenience sampling from local communities in the Republic of Korea. Participants completed both a text-based and a color-based Stroop task on a tablet. Cognitive load was indexed using prefrontal functional near-infrared spectroscopy (fNIRS), measuring functional connectivity (FC) and global network efficiency. Behavioral outcome measures included accuracy, reaction time, and the composite efficiency score. Clinical validity was assessed by correlating task performance with parent-reported attention problems using a standardized behavioral rating scale. General cognitive ability was controlled using a standardized intelligence measure. Condition differences were analyzed using paired-sample *t* tests (α=.05), clinical associations using Pearson correlations with Fisher *r*-to-*z* transformation, and predictor contributions using random forest regression.

**Results:**

In the paired-sample *t* tests, the color-based format yielded significantly higher accuracy (0.91 vs 0.86; mean difference 0.05, 95% CI 0.03-0.08; t_126_=3.81, Cohen *d*=0.34, *P*<.001), faster reaction times (1183 vs 1269 ms; mean difference −85.4 ms, 95% CI −107.2 to −63.5; t_126_=−7.72, Cohen *d*=–0.69, *P*<.001), and superior composite performance (t_126_=6.81, Cohen *d*=0.62, *P*<.001). Pearson correlations revealed that color-based performance was significantly associated with parent-reported attention problems (*r*=−0.20, 95% CI −0.37 to −0.03; *P*=.03), whereas text-based performance was not (*r*=−0.05, 95% CI −0.23 to 0.12;’ *P*=.56); the Fisher *r*-to-*z* test indicated this difference was not statistically significant (*z*=1.54, *P*=.12). fNIRS-derived neural indices (ΔFC, global efficiency) did not differ significantly between conditions (*P*>.05). Random forest analyses indicated that after controlling for age and general cognitive ability, individual variations in prefrontal efficiency accounted for 40% to 44% of residual performance variance.

**Conclusions:**

This study provides some of the first empirical evidence that the interaction format substantially influences task demands and measurement validity in pediatric digital assessments, extending cognitive load theory into digital health assessment design. Color-based response formats that minimize extraneous semantic processing yield superior performance and stronger clinical validity compared to text-based formats. These findings suggest that prioritizing response modalities aligned with children’s developmental capabilities may improve the clinical utility of digital attention assessments.

## Introduction

Digital health technologies have rapidly expanded in recent years [1], especially in mental health applications that offer both assessment and intervention capabilities [2-4]. One of the advantages driving this growth is accessibility: digital health tools can be accessed on demand through widely available consumer devices, such as tablets and smartphones [5,6]. However, this accessibility also presents a challenge. Because these tools are often designed for self-guided use on personal devices, sustained user engagement becomes critical for their effectiveness [7,8]. Without adequate engagement, users may discontinue assessments prematurely or fail to complete intervention protocols, undermining potential clinical benefits [9,10]. Consequently, strategies such as gamification, which incorporate game-like elements to enhance motivation and sustain participation [11], have emerged as important approaches in digital health applications [12]. Within this framework, specific design decisions, such as interaction formats, are important considerations, yet empirical evidence on how these decisions specifically impact clinical outcomes is still emerging [13,14].

Although the interaction format is often discussed in terms of usability and user experience, its implications extend beyond engagement alone. The way tasks are designed and presented directly influences the cognitive demands placed on users, which, in turn, affects both measurement accuracy and clinical validity [13]. For instance, the visual appearance of an avatar in a therapeutic virtual environment can shape user trust and therapeutic alliance [15], while real-time biofeedback mechanisms can simultaneously enhance motivation through gamification principles and improve clinical performance by enabling timely intervention [16,17]. However, design decisions can also degrade performance when they fail to account for the cognitive demands placed on users [18]. This is particularly critical when the target population includes individuals with limited cognitive resources, such as children or older adults, who are more vulnerable to the effects of extraneous cognitive demands [19-21]. In such cases, poorly calibrated design choices can introduce systematic bias, degrade measurement fidelity, and compromise the validity of clinical assessments [22].

Cognitive load, the mental effort required to process information and execute tasks [23,24], is substantially influenced by design choices. This relationship has been extensively studied in educational and e-learning contexts, where optimizing information presentation is essential for learning outcomes [25]. Research in this domain consistently demonstrates that appropriate modality selection and content structure reduce cognitive load and improve performance. For example, visual presentations combined with narration outperform voice-only formats, and temporally organized content enhances effectiveness in mobile learning [26,27]. These findings underscore that cognitive load is not solely determined by task content but also substantially shaped by how information is presented and how users interact with it [28,29]. Consequently, failing to optimize interaction formats in digital assessments may lead to “noise” in the data, where performance reflects interface-induced load rather than the target clinical construct. However, despite the clear relevance of these principles to digital health, empirical studies examining cognitive load in this domain have focused primarily on clinical workflows and professional users [30,31], while patient-facing assessment and intervention tools have received comparatively less attention. This gap is consequential because elevated cognitive load in assessment contexts can inflate error rates, slow response times, and reduce completion rates, thereby introducing confounding variables that mask a child’s true cognitive ability. Addressing this issue is therefore essential for designing digital assessment tools that are not only developmentally appropriate but also clinically valid, particularly for pediatric populations.

Building on this background, this study investigated how variations in the interaction format affect cognitive load and performance validity in a tablet-based Stroop task for children. The Stroop task, one of the most widely used paradigms for assessing selective attention and inhibitory control [32], provides an ideal model for examining how the interaction format influences cognitive load. In its classic form, participants name the ink color of a color word while ignoring its meaning, with performance typically faster and more accurate when color and meaning match (congruent trials) and slower with more errors when they conflict (incongruent trials) [33]. This performance difference reflects the cognitive effort required to resolve interference, making the paradigm particularly sensitive to design-induced variations in mental demands [34].

To investigate how these design variations affect both cognitive demands and measurement validity, we compared two response formats in a tablet-based Stroop task for children: selecting written color-word labels (text condition), which preserves semantic conflict, versus selecting color patches (color condition), which eliminates semantic processing demands. This distinction is particularly relevant for pediatric populations, in whom reading is not yet fully automated and text-based response options may conflate reading fluency with inhibitory control [35]. Cognitive load was measured using prefrontal functional near-infrared spectroscopy (fNIRS), a noninvasive neuroimaging method well suited for monitoring attentional effort in interactive tasks with children, alongside behavioral measures of accuracy and reaction time [36].

Through this approach, the study addressed two primary research questions:

- Does the interaction format (text-based vs color-based response selection) affect cognitive load and behavioral performance?
- If so, do these performance differences translate into differential clinical validity, as measured by associations with standardized attention assessments?

By addressing these questions, the study aimed to underscore the importance of interaction format calibration in pediatric digital assessments. In addition, it aimed to provide empirical guidance for designing Stroop-based attention tools that are both developmentally appropriate and clinically valid for children.

## Methods

### Participants

#### Inclusion and Exclusion Criteria

The study recruited typically developing children aged 6-12 years, a developmental period during which attentional control is maturing and cognitive resources remain limited compared to adults [37]. Inclusion criteria included (1) no history of psychiatric, neurological, or developmental disorders; (2) no visual or color vision impairments; and (3) sufficient language and communication abilities to follow instructions and complete the task. Children who did not meet any of these criteria were excluded from participation.

#### Sampling Procedures

Participants were recruited through convenience sampling via local community advertisements in the Republic of Korea between January and September 2023. A total of 137 children were initially enrolled. Of these, 7 (5.1%) were excluded due to technical issues during data acquisition (eg, cloud transfer failure, fNIRS calibration failure), and 3 (2.2%) withdrew during the session. The final analytic sample comprised 127 (92.7%) children who participated in this within-subjects comparison design.

#### Participant Characteristics

Typically developing children were selected to establish a baseline effect of the interaction format on cognitive load and task performance without the confounding influences of clinical symptoms [37]. This sample ensured sufficient developmental readiness to perform tablet-based tasks, while capturing meaningful individual variation in attentional functioning.

#### Sample Size, Power, and Precision

The sample size was determined by the available participant pool during the recruitment period. A sensitivity analysis (paired-sample *t* test, 2-tailed, α=.05, power=0.80, n=127) indicated that the study was powered to detect effects of Cohen *d*≥0.25, corresponding to small-to-medium effects. This threshold is below or comparable to effect sizes typically reported in pediatric Stroop studies [38], suggesting adequate statistical power for the primary within-subject comparisons.

### Task and Procedure

#### Stroop Task Design

**Figure 1:**
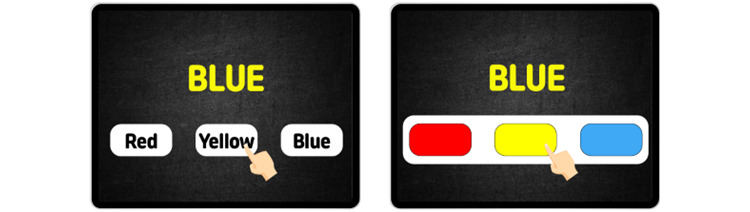
Stroop task conditions used in a within-subject comparison of interaction formats among 127 typically developing children aged 6-12 years. (Left) Text-based response condition, where participants identified the ink color by selecting a written label. (Right) Color-based response condition, where participants identified the ink color by selecting the corresponding color patch.

The experiment was conducted on a tablet (Samsung Galaxy Tab Advanced2) and implemented two Stroop-based tasks that systematically manipulated the interaction format (Figure 1). Both tasks presented participants with color words displayed in incongruent ink colors (eg, the word “blue” written in yellow ink), requiring participants to identify the ink color while ignoring the word meaning. This core Stroop conflict was preserved across both conditions to maintain sensitivity to attentional control and inhibitory processes.

In the *text condition*, participants identified the ink color by selecting from among written color-word labels (eg, “Yellow,” “Red,” “Blue”). This format preserved the traditional Stroop paradigm and maintained the semantic conflict central to clinical and attentional assessment contexts [39,40]. The automatic tendency to process word meaning directly competed with the controlled requirement to report the ink color, requiring active suppression of irrelevant semantic processing and elevating cognitive load through inhibitory control and conflict resolution [41-43].

In the *color condition*, participants identified the ink color by selecting a color patch instead of a written label. This format retained the core Stroop conflict, as the stimulus still presented a mismatch between word meaning and ink color. However, it eliminated the additional layer of interference arising from competing verbal response options [44]. By removing word-word competition at the response stage, this condition aimed to reduce unnecessary cognitive demands, while preserving the attentional control requirements central to the Stroop effect [45,46]. This manipulation reflects how interface elements, such as response modality, can be deliberately adjusted to calibrate cognitive demands for specific populations in digital assessment contexts. Within the cognitive load theory framework [24], this design reflects the distinction between intrinsic and extraneous loads. The core Stroop conflict between word meaning and ink color constitutes the intrinsic load, which was held constant across both conditions. The response format manipulation targeted the extraneous load: the text condition introduced additional semantic processing at the response stage, whereas the color condition eliminated this secondary interference.

To minimize extraneous influences unrelated to interaction format manipulation, both conditions used identical visual presentation parameters (font size, button size, layout) and response mechanics (touch-based selection). The two conditions differed only in the format of response options provided to participants.

#### Experimental Protocol

**Figure 2:**
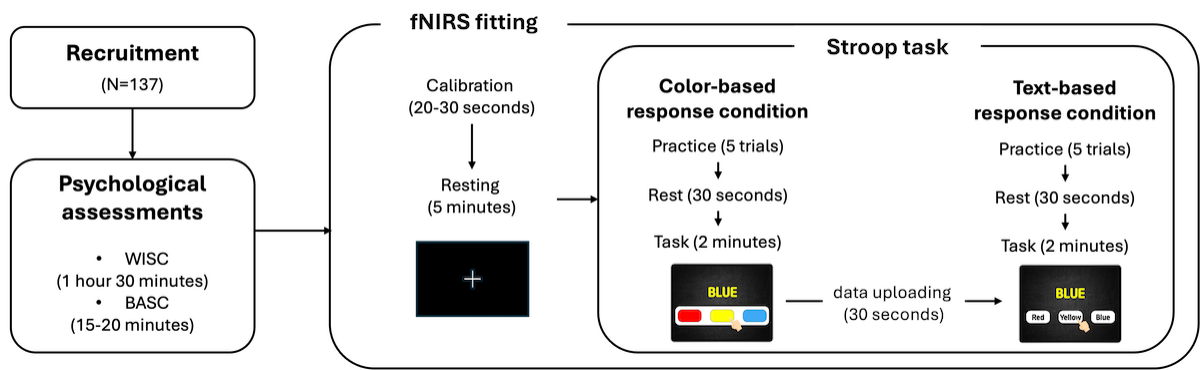
Overview of the experimental procedure for a within-subject comparison of two Stroop task response formats in 127 typically developing children aged 6-12 years. Participants completed standardized cognitive (K-WISC-V) and behavioral (BASC-2) assessments, followed by fNIRS device fitting, a 5-minute resting-state baseline, and two Stroop task conditions (color-based response and then text-based response, 45 trials each) separated by a 30-second rest interval. BASC-2: Behavior Assessment System for Children, Second Edition; fNIRS: functional near-infrared spectroscopy; K-WISC-V: Korean version of the Wechsler Intelligence Scale for Children, Fifth Edition.

All participants completed the study following a standardized protocol, as shown in Figure 2. Upon arrival, participants first completed standardized assessments of cognitive ability (intelligence quotient [IQ]) and behavioral attention. These assessments characterized individual differences in cognitive ability and attentional functioning, allowing control for these factors in subsequent analyses.

Following the assessments, participants were fitted with the fNIRS device, and a brief calibration was performed to ensure signal quality. A 5-minute resting-state baseline was then recorded while participants fixated on a white cross presented on a black background under quiet, dimly lit conditions. This baseline served as a reference for subsequent task-related neural activity.

After the baseline recording, participants performed both Stroop tasks. The color condition was administered first, followed by the text condition. Although randomization is standard, this fixed order was intentionally chosen to provide a scaffolding effect for the pediatric participants [47], ensuring task comprehension with the more intuitive format before introducing the higher linguistic demand of the text condition. To account for potential order effects, we incorporated a rest period and treated performance as a within-subject comparison.

Each task began with five practice trials to ensure comprehension. Participants could repeat the practice block if they required additional familiarization. Following a 30-second rest period, the main task block was presented, consisting of 45 trials per condition. Accuracy (correct/incorrect) and reaction time (time from stimulus onset to response) were recorded for each trial. Trial presentation was self-paced, with the next trial appearing immediately after a response was registered. A 30-second interval separated the two task conditions to allow data uploading and provide a brief rest.

All procedures were conducted in a quiet, controlled laboratory environment to minimize distractions and ensure consistent conditions across participants. The entire session, including assessments, device setup, baseline recording, and task performance, lasted approximately 90 minutes.

### Data Acquisition

#### Psychometric Assessments

To control for individual differences and ensure reliable interpretation of task performance, multiple standardized measures were used.

General cognitive ability was assessed using the Korean version of the Wechsler Intelligence Scale for Children, Fifth Edition (K-WISC-V) [48], a widely used and validated measure of intelligence in children [49]. Administration was carried out by a licensed clinical psychologist following standardized procedures. The assessment yielded five primary index scores: the Verbal Comprehension Index (VCI), Visual Spatial Index (VSI), Fluid Reasoning Index (FRI), Working Memory Index (WMI), and Processing Speed Index (PSI). From these indices, a full-scale intelligence quotient (FSIQ) score was derived, providing a comprehensive measure of general intellectual functioning. IQ assessment was included to characterize the cognitive profile of the sample and to control for potential variance in Stroop performance.

Attention-related functioning was evaluated using the Korean version of the Behavior Assessment System for Children, Second Edition (BASC-2) [50], a comprehensive and validated measure of children’s behavioral and emotional functioning [51]. The parent-report form was used to obtain ecologically valid observations of children’s everyday attentional behaviors. The BASC-2 consists of 160 items rated on a 4-point Likert scale (“never” to “almost always”), yielding composite and subscale scores. For this analysis, we focused on the Attention Problems subscale, which captures tendencies such as distractibility and difficulty sustaining focus, providing a behavioral index of attentional control in real-world contexts [52]. In contrast to the Stroop task, which reflects laboratory performance, BASC-2 ratings capture everyday attentional functioning, providing a complementary perspective for evaluating the clinical relevance of different interaction formats. This dual perspective allowed examination of whether different interaction formats show differential associations with standardized measures of attention, serving as a criterion for evaluating clinical relevance. T scores from the Attention Problems subscale as the primary indicators of behavioral attentional functioning in all analyses.

#### Neuroimaging

Cortical activation during task performance was measured using fNIRS, a noninvasive neuroimaging method that detects changes in oxygenated and deoxygenated hemoglobin concentrations in the cortex. fNIRS was selected for its robustness against motion artifacts, ecological suitability for interactive tablet-based tasks, and safety for pediatric populations [53,54].

The NIRSIT Lite device (OBELAB) was used, providing 15 measurement channels covering the prefrontal cortex (PFC). The PFC is closely associated with attentional control, working memory, and inhibitory processes underlying cognitive load [55,56]. The device operates with near-infrared light at wavelengths of 780 and 850 nm, with intensities below 5 mW to ensure participant safety. The optode array was specifically designed to accommodate the smaller head sizes typical of children, with source-detector distances of ~3 cm to maximize cortical sensitivity, while maintaining signal quality.

The device was secured on participants’ foreheads using an adjustable headband, positioned to cover bilateral prefrontal regions. Following placement, a brief calibration procedure was performed to verify signal quality across all channels. Signal quality was evaluated using a signal-to-noise (SNR) threshold of 30 dB; channels falling below this threshold were to be excluded from analysis. All channels met the quality threshold across all participants. At the time point level, data points with SNR *z* scores exceeding 2.58 were identified as outliers and removed prior to connectivity computation. Continuous recording of hemodynamic responses was maintained throughout the resting-state baseline and both Stroop task conditions, enabling quantification of task-related prefrontal activation as an objective index of cognitive effort. Data were sampled at 8.138 Hz and stored for offline preprocessing and analysis.

### Analytic Strategy

#### Behavioral Data

Data from the paper-based assessments (K-WISC-V and BASC-2) were scored according to the standardized procedures provided in each manual. For BASC-2, T scores from the Attention Problems subscale were used as indicators of behavioral attentional functioning. Behavioral performance on the Stroop task was quantified using accuracy (proportion of correct responses) and reaction time (mean response latency in milliseconds) for each condition. To account for differences in trial completion and speed-accuracy trade-offs, a composite performance score was calculated as follows:



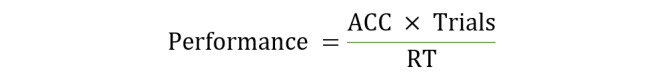



where ACC refers to accuracy and RT refers to reaction time.

This composite index is conceptually equivalent to the Rate Correct Score [57], providing an integrated measure of task efficiency that accounts for potential speed and accuracy trade-offs. Paired-sample *t* tests were conducted to compare accuracy, reaction time, and composite performance between the color and text conditions, as all participants completed both tasks. Effect sizes were calculated using Cohen d for paired samples.

#### Neuroimaging Data

Functional connectivity (FC) and global efficiency were selected as neural indices because the study aimed to assess PFC network–level organization during sustained task performance rather than localized hemodynamic responses. Global efficiency provides an index of how efficiently information is integrated across the PFC network and has been shown to relate to the cognitive load burden in fNIRS studies [58]. Prefrontal fNIRS data were preprocessed to obtain estimates of the FC. Resting-state connectivity was computed as Pearson correlation coefficients between all pairs of fNIRS channels and then transformed using Fisher *Z* transformation to stabilize variance and approximate normality [59]. Prior to connectivity analysis, raw optical density data were motion-corrected using a moving SD approach and band-pass-filtered (0.005-0.1 Hz) to remove physiological noise, following standard fNIRS preprocessing procedures [60]. Task-related connectivity during each Stroop task was calculated using the same procedure. Cognitive activation was operationalized as the difference between task-related and resting-state connectivity as follows:



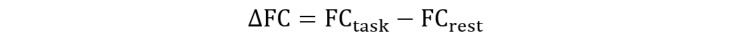



This subtraction isolates connectivity changes specifically attributable to task engagement, controlling for baseline individual differences in PFC network strength. In addition to connectivity strength, global efficiency was computed from task-related connectivity matrices using graph-theoretical analysis. Global efficiency is defined as the average inverse shortest path length across nodes in a network and provides an index of how efficiently information can be exchanged across the PFC network [61]. Higher global efficiency indicates more economical neural organization, whereas lower efficiency suggests greater cognitive demands and less efficient processing [58]. Together, ∆FC and global efficiency provided complementary measures of PFC network dynamics during task performance. To examine condition differences in these neural indices, paired-sample *t* tests were conducted comparing ΔFC and global efficiency between the color and text conditions.

#### Clinical Correlation Analysis

To evaluate the clinical relevance of each interaction format, Pearson correlations were computed between BASC-2 Attention Problems T scores and task performance indices (accuracy, reaction time, composite performance) in each condition. Fisher *r*-to-*z* transformation was applied to test whether correlation coefficients differed significantly between the two conditions. Stronger correlations with standardized behavioral measures indicated greater clinical validity for a given interaction format.

#### Predictive Modeling

Random forest regression models were used to examine the relative contribution of behavioral, neural, and psychometric measures in predicting Stroop task performance. A random forest was selected for several reasons. First, the dataset included heterogeneous predictors spanning behavioral, neural, and psychometric domains, which are unlikely to conform to linear assumptions [62]. Second, a random forest offers stability with a modest sample size by aggregating predictions across multiple decision trees through bootstrap resampling, reducing overfitting risk [63]. Third, a random forest provides variable importance estimates, enabling assessment of the relative influence of different predictor types [64].

All predictor variables, including task performance measures (accuracy, reaction time, composite performance), neural indices (∆FC, global efficiency), and psychometric scores (FSIQ, BASC-2 Attention Problems T scores), were entered into the models. The models were fitted separately for each condition (color and text) to examine whether the importance of neural versus behavioral predictors varies across interaction formats.

To control for potential confounding by age and general cognitive ability, additional models were fitted using residualized outcome measures. Specifically, age and the FSIQ were regressed out of the composite performance measure using linear regression, and the resulting residuals were used as dependent variables in supplementary random forest models. This approach ensured that observed predictor effects were not attributable to basic demographic or intellectual factors.

Model performance was evaluated using the out-of-bag (OOB) prediction error, which provides an unbiased estimate of generalization performance without requiring a separate validation set. Variable importance was assessed using the mean decrease in accuracy, which quantifies the reduction in model accuracy when a given predictor is randomly permuted. All random forest models were implemented using *scikit-learn* in Python with 500 trees with default hyperparameters (mtry=p/3 for regression, where p is the number of predictors).

### Ethical Considerations

All procedures involving human participants were conducted in accordance with the ethical standards of the Institutional Research Committee and with the Declaration of Helsinki. The study protocol was reviewed and approved by the Institutional Review Board (IRB) of the Korea Brain Research Institute (approval number: KBRI-202103-HR-02). Written informed consent was obtained from the legal guardians of all child participants prior to enrollment, and verbal assent was obtained from the children using developmentally appropriate language. Participants and their families were informed of their right to withdraw from the study at any time without penalty. Guardians received KRW 30,000 (~US $22) as compensation for participation and transportation expenses, and child participants received a small gift valued at ~KRW 20,000 (~US $15). All collected data were deidentified and stored securely to ensure confidentiality and privacy, and no identifiable images of participants were collected.

## Results

**Sample Characteristics and Descriptive Statistics**

**Figure 3:**
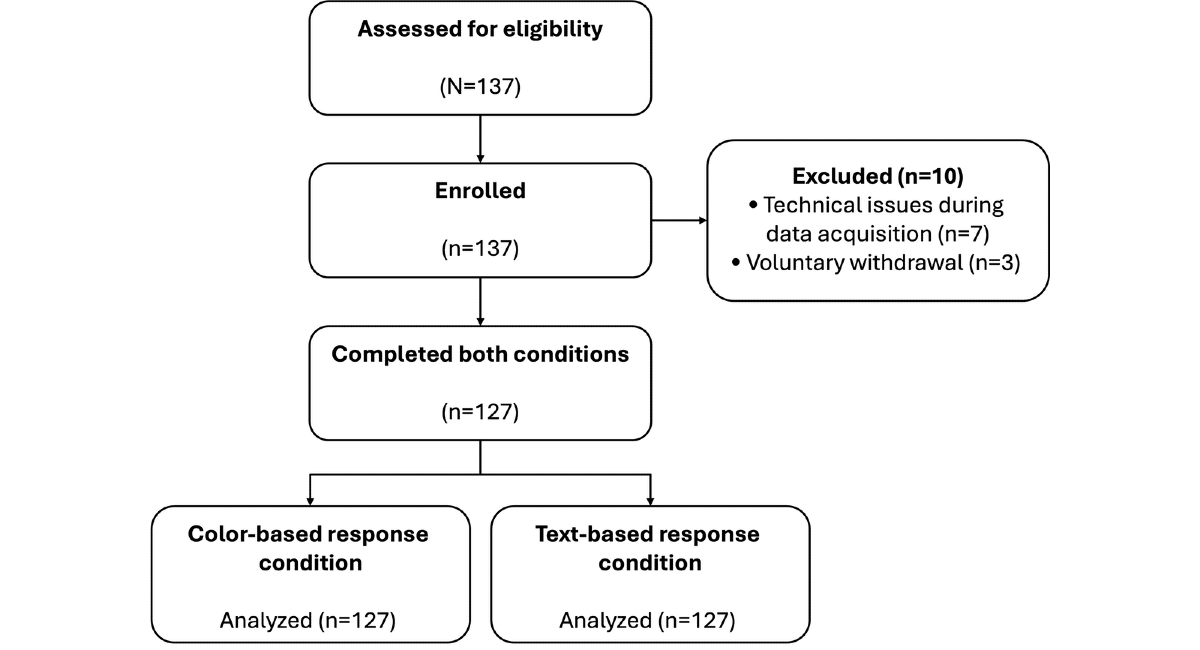
Participant flowchart depicting enrollment, exclusions, and final analytic sample in a within-subject comparison of two Stroop task response formats (color-based vs text-based response) among typically developing children aged 6-12 years. Of 137 children assessed for eligibility, 127 (92.7%) completed both tasks and were included in the final analyses.

The flow of participants through each stage of the study is depicted in Figure 3. The final sample comprised 127 children (n=55, 43.3%, girls; n=72, 56.7%, boys; mean age 9.15, SD 1.56 years, range 6-12 years).

Detailed descriptive results are provided in Table 1. All primary analysis variables had complete data for all participants; no missing data imputation was required. On the K-WISC-V, the mean FSIQ was 105.71 (SD 12.08), with subscale scores within the normative range. The Attention Problems subscale of the BASC-2 yielded a mean T score of 46.76 (SD 9.15), also within the normative range.

**Table 1:** Descriptive statistics, paired-sample t test comparisons, and Pearson correlations with BASC-2^a^ Attention Problems T scores for typically developing children (n=127) aged 6-12 years who completed two Stroop tasks (color-based vs text-based response) in a within-subject design.

Measure and conditions			Mean (SD)		*t* (*df*)*/*Pearson correlation coefficient, r		*P* value		Cohen *d*
Age (years)			9.15 (1.56)		—^b^		—		—
**Index**									
	FSIQ^c^ (K-WISC-V^d^)	105.71 (12.08)		—		—		—	
	VCI^e^ (K-WISC-V)	105.91 (13.92)		—		—		—	
	VSI^f^ (K-WISC-V)	103.92 (13.88)		—		—		—	
	FRI^g^ (K-WISC-V)	104.66 (14.19)		—		—		—	
	WMI^h^ (K-WISC-V)	102.47 (12.42)		—		—		—	
	PSI^i^ (K-WISC-V)	105.20 (12.43)		—		—		—	
Attention problems (BASC-2)			46.76 (9.15)		-		—		—
**Accuracy**									
	Color	0.91 (0.09)		t_126_=3.81		<.001		0.34	
	Text	0.86 (0.16)		—		—		—	
**Reaction time (ms)**									
	Color	1183.21 (146.78)		t_126_=–7.72		<.001		-0.69	
	Text	1268.56 (145.18)		—		—		—	
**Performance**									
	Color	0.036 (0.007)		t_126_=6.81		<.001		0.62	
	Text	0.031 (0.008)		—		—		—	
∆**FC**^j^									
	Color	0.12 (0.33)		t_126_=1.65		.10		0.15	
	Text	0.07 (0.29)		—		—		—	
**Prefrontal global efficiency**									
	Color	0.55 (0.17)		t_126_=1.52		.13		0.14	
	Text	0.53 (0.17)		—		—		—	
**Attention × performance**									
	Color	—		*r*=–0.20		.03		—	
	Text	—		*r*=–0.05		.56		—	

^a^BASC-2: Behavior Assessment System for Children, Second Edition.

^b^Not applicable.

^c^FSIQ: full-scale intelligence quotient.

^d^K-WISC-V: Korean version of the Wechsler Intelligence Scale for Children, Fifth Edition.

^e^VCI: Verbal Comprehension Index.

^f^VSI: Visual Spatial Index.

^g^FRI: Fluid Reasoning Index.

^h^WMI: Working Memory Index.

^i^PSI: Processing Speed Index.

^j^FC: functional connectivity.

In the Stroop task, accuracy was higher in the color condition (mean 0.91, SD 0.09) than in the text condition (mean 0.86, SD 0.16). Reaction times were also shorter in the color condition (mean 1183.21, SD 146.78 ms) compared to the text condition (mean 1268.56, SD 145.18 ms). Composite performance was correspondingly greater in the color condition (mean 0.036, SD 0.007) than in the text condition (mean 0.031, SD 0.008), reflecting more efficient task execution overall.

**Figure 4:**
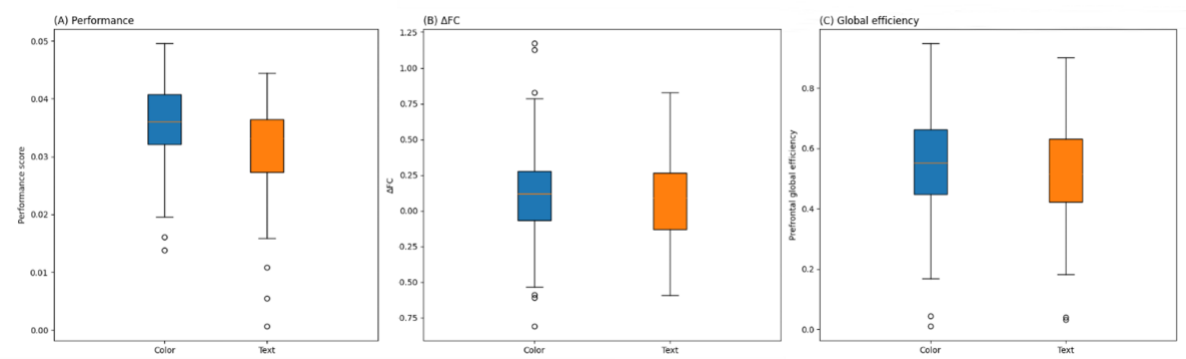
Boxplots comparing Stroop task indices between color-based and text-based response formats in 127 typically developing children aged 6-12 years (within-subject design). (A) Performance score, (B) task-evoked connectivity change (ΔFC), and (C) prefrontal global efficiency. FC: functional connectivity.

Neuroimaging measures derived from prefrontal fNIRS data showed parallel patterns. Task-related FC (Fisher *Z* transformed) was numerically higher in the color condition (mean 0.81, SD 0.28) than in the text condition (mean 0.76, SD 0.24). When resting-state connectivity was subtracted to isolate task-specific change ∆FC, values were again greater in the color condition (mean 0.12, SD 0.33) than in the text condition (mean 0.07, SD 0.29). Prefrontal global efficiency was modestly higher in the color condition (mean 0.55, SD 0.17) than in the text condition (mean 0.53, SD 0.17). All three neural indices consistently showed numerically higher values in the color condition. For visualization of distributional patterns across conditions, boxplots of performance, ∆FC, and prefrontal global efficiency are shown in Figure 4.

### Condition Comparisons and Correlations

Paired-sample *t* tests indicated significant condition differences for all three behavioral measures. Accuracy was significantly higher in the color condition (t_126_=3.81, *d*=0.34, 95% CI 0.03-0.08; *P*<.001). Reaction time was significantly shorter in the color condition (t_126_=–7.72, *d*=–0.69, 95% CI −107.2 to −63.5; *P*<.001). Composite performance was significantly better in the color condition (t_126_=6.81, *d*=0.62, 95% CI 0.003-0.006; *P*<.001). Effect sizes ranged from medium to large, suggesting that the interaction format had a more pronounced impact on manifest behavior than on observable neural network changes within this sample size. In contrast, neural indices showed no reliable differences between conditions. The task-related FC did not differ significantly (t_126_=1.65, *d*=0.15, 95% CI −0.01 to 0.10; *P*=.10). Similarly, ∆FC (t_126_=1.65, *d*=0.15, 95% CI −0.01 to 0.10; *P*=.10) and prefrontal global efficiency (t_126_=1.52, *d*=0.14, 95% CI −0.01 to 0.05; *P*=.13) showed no significant differences, although all trended in the direction of higher values in the color condition.

Correlational analyses with BASC-2 Attention Problems T scores, a validated behavioral measure of attentional difficulties, showed that higher scores (indicating more attention problems) were associated with lower composite performance in the color condition (*r*=−0.20, 95% CI −0.37 to −0.03; *P*=.03), whereas the corresponding performance in the text condition was not significantly related (*r*=−0.05, 95% CI −0.23 to 0.12; *P*=.56). Fisher *r*-to-*z* transformation indicated that this difference approached but did not reach conventional significance (*z*=1.54, *P*=.12). Neural indices showed weak and nonsignificant correlations with attention problems in both conditions: color ∆FC (*r*=−0.07, 95% CI −0.24 to 0.10; *P*=.42), color global efficiency (*r*=−0.05, 95% CI −0.23 to 0.12; *P*=.55), text ∆FC (*r*=–0.14, 95% CI −0.31 to 0.03; *P*=.10), and text global efficiency (*r*=0.03, 95% CI −0.14 to 0.20; *P*=.73). Complete correlation coefficients are provided in Table 1.

### Random Forest Feature Importance

To examine which features contributed to individual differences in performance within each interaction format, we estimated contributions using condition-specific random forest models with permutation-based variable importance (reported as normalized percentages; see the *Methods* section for details). Variables with higher importance scores contributed more to reducing the prediction error across decision trees.

In the color condition, age showed the largest contribution (58.86%), followed by cognitive measures including the PSI (17.06%) and the FSIQ (10.25%). Smaller contributions came from BASC-2 Attention Problems (4.79%), gender (4.75%), and neural indices (∆FC: 2.82%; global efficiency: 1.47%). The model achieved a cross-validated *R*^2^ of 0.091, corresponding to Δ*R*^2^=0.100 relative to a mean-only baseline. Full importance values are provided in Table 2.

**Table 2:** Random forest permutation importance by condition for typically developing children (n=127) aged 6-12 years completing two Stroop tasks (within-subject design; strict models^a^).^b^

Condition and features		Mean_perm_^c^ (SD) across validation folds	Normalized importance score (%)^d^
**Color-based response**			
	Age (years)	0.160 (0.036)	58.86
	PSI^e^ (K-WISC-V^f^)	0.046 (0.025)	17.06
	FSIQ^g^ (K-WISC-V)	0.028 (0.047)	10.25
	Attention problems (BASC-2^h^)	0.013 (0.030)	4.79
	Gender	0.013 (0.008)	4.75
	∆FC^i^	0.008 (0.050)	2.82
	Global efficiency (prefrontal)	0.004 (0.081)	1.47
**Text-based response**			
	Age (years)	0.375 (0.184)	73.21
	PSI (K-WISC-V)	0.044 (0.065)	8.53
	∆FC	0.033 (0.024)	6.34
	Global efficiency (prefrontal)	0.023 (0.008)	4.40
	Gender	0.018 (0.015)	3.51
	FSIQ (K-WISC-V)	0.014 (0.044)	2.78
	Attention problems (BASC-2)	0.004 (0.018)	0.77
	WMI^j^ (K-WISC-V)	0.002 (0.039)	0.47

^a^Strict models include all demographic, psychometric, and neural predictors.

^b^Features are ranked by normalized importance.

^c^Mean_perm_: mean permutation importance across bootstrap samples. Higher values indicate greater contribution to model prediction.

^d^Normalized percentages add up to 100% within each condition.

^e^PSI: Processing Speed Index.

^f^K-WISC-V: Korean version of the Wechsler Intelligence Scale for Children, Fifth Edition.

^g^FSIQ: full-scale intelligence quotient.

^h^BASC-2: Behavior Assessment System for Children, Second Edition.

^i^FC: functional connectivity.

^j^WMI: Working Memory Index.

In the text condition, age again dominated (73.21%), with contributions from the PSI (8.53%), neural indices (∆FC: 6.34%; global efficiency: 4.40%), gender (3.51%), and the FSIQ (2.78%). BASC-2 Attention Problems (0.77%), and the WMI (0.47%) contributed minimally. The model achieved a cross-validated *R*^2^ of 0.233, corresponding to ∆*R*^2^=0.274 relative to baseline.

Because age and the FSIQ contributed strongly in the original models, we conducted supplementary analyses using residualized outcome measures. Age and the FSIQ were first regressed out of composite performance (color: *R*^2^=0.271; text: *R*^2^=0.413), and the resulting residuals were used as dependent variables in new random forest models with neural indices and BASC-2 Attention Problems as predictors.

In the residualized color condition model, neural indices emerged as the largest contributors: global efficiency (39.79%) and ∆FC (35.75%), followed by BASC-2 Attention Problems (24.46%). In the residualized text condition model, a similar pattern emerged: global efficiency (43.60%), ∆FC (33.02%), and BASC-2 Attention Problems (23.37%). Residualized models achieved cross-validated *R*^2^ values of 0.113 (color) and 0.202 (text), indicating that after controlling for age and general cognitive ability, neural and behavioral attention measures accounted for 11% to 20% of the remaining variance in performance. Complete residualized importance values are provided in Table 3.

**Table 3:** Random forest permutation importance by condition for typically developing children (n=127) aged 6-12 years completing two Stroop tasks (within-subject design; residualized models^a^, with age and FSIQ^b^ regressed out).^c^

Condition and features		Mean_perm_^d^ (SD) across validation folds	Normalized importance score (%)^e^
**Color-based response**			
	Global efficiency (prefrontal)	0.605 (0.044)	39.79
	∆FC^f^	0.543 (0.050)	35.75
	Attention problems (BASC-2^g^)	0.372 (0.038)	24.46
**Text-based response**			
	Global efficiency (prefrontal)	0.640 (0.067)	43.60
	∆FC	0.484 (0.048)	33.02
	Attention problems (BASC-2)	0.343 (0.037)	23.37

^a^Residualized models control for age and the full-scale intelligence quotient.

^b^FSIQ: full-scale intelligence quotient.

^c^Features are ranked by normalized importance.

^d^Mean_perm_: mean permutation importance across bootstrap samples. Higher values indicate greater contribution to model prediction.

^e^Normalized percentages add up to 100% within each condition.

^f^FC: functional connectivity.

^g^BASC-2: Behavior Assessment System for Children, Second Edition.

## Discussion

### Principal Findings

This study examined how the interaction format influences cognitive load and measurement validity in a tablet-based Stroop task for children. Using within-subject comparisons between color-based and text-based response formats, combined with prefrontal fNIRS and clinical behavioral measures, we addressed two primary questions: whether the interaction format affects cognitive load and performance and whether performance differences translate into differential clinical validity.

Behavioral results clearly demonstrated the effects of interaction formats. The color-based response condition yielded significantly faster responses, higher accuracy, and better composite performance compared to the text-based response condition (all *P*<.001, with medium-to-large effect sizes). Critically, color-based performance showed a significant correlation with parent-reported attention problems (BASC-2; *r*=−0.20, *P*=.03), whereas text-based performance did not (*r*=−0.05, *P*=.56). Although the relatively wide CIs suggest that the precise magnitude of these associations warrants replication, this differential pattern indicates that the color format showed a pattern more consistent with clinical validity by better isolating attentional control from other cognitive demands. Random forest analyses further revealed that after controlling for age and general cognitive ability, neural indices of prefrontal efficiency (global efficiency, ∆FC) emerged as the dominant predictors of individual performance differences in both formats, accounting for ~35% to 44% of residual variance.

The observed performance differences are consistent with the intended manipulation of cognitive load. The text-based response format required dual semantic processing: participants had to ignore the word meaning while simultaneously mapping the ink color to a written label [33]. In contrast, the color-based response format preserved the core Stroop conflict while eliminating competing verbal response options, thereby reducing extraneous cognitive load [45]. Consistent with this interpretation, the color condition showed not only superior behavioral performance but also descriptively higher prefrontal efficiency (∆FC, global efficiency). Although these neural differences did not reach statistical significance, such small-to-moderate effects are consistent with the typical magnitude observed in fNIRS research with developmental populations [65]. In addition, PFC maturation continues throughout childhood, and the developing PFC networks in our sample may not yet exhibit sufficiently stable connectivity patterns to produce detectable condition-level differences [66]. Furthermore, because both conditions shared the core Stroop conflict and differed only in the response format, the neural workload differences may have been too subtle to detect at the group level.

It is worth noting that modifying the response format may have also altered the relative contribution of different cognitive processes at the response selection stage. However, the preservation of the core Stroop conflict at the stimulus level and the significant association between color-based performance and clinical attention measures suggest that the fundamental inhibitory control construct was maintained across both formats. Moreover, color-based formats may be especially appropriate for children in early literacy stages, for whom text-based responses could introduce construct-irrelevant variance related to reading ability rather than attentional control [35].

As an exploratory analysis, random forest analyses provided a complementary view of how individual differences relate to task performance in each format. In the strict models, age had the largest contribution in both formats, and cognitive measures, such as the PSI and FSIQ, accounted for much of the remaining signal, with neural features (prefrontal global efficiency, ∆FC) contributing smaller percentages (Table 2). Because age and IQ dominated these models, we conducted residualized analyses that removed their variance from the outcome prior to modeling. In these residualized models, neural indices carried most of the remaining predictive signal in both formats, with BASC-2 Attention Problems accounting for the remainder (Table 3).

These findings highlight two key points. First, the higher contribution of BASC-2 Attention Problems in the color condition is consistent with the stronger correlation observed between color-based performance and the clinical attention benchmark, suggesting that the lower-load interface better isolates attentional control from other developmental influences. Second, the dominant role of age and IQ in the strict models underscores the need to treat these variables as covariates in attentional research, echoing prior findings that developmental and general cognitive factors exert a substantial influence on performance [35,67]. Notably, however, the relative contribution of age was smaller in the color format (58.86%) than in the text format (73.21%). This pattern suggests that reducing semantic interference was associated with lower cognitive load indicators and a smaller relative contribution of developmental differences dominating performance, thereby providing a clearer assessment of attentional control.

At the same time, format-specific nuances emerged that distinguished average performance from individual variability. In terms of mean values, both ∆FC and global efficiency were descriptively higher in the color condition, which is consistent with more efficient prefrontal organization when cognitive demands are lower [61]. However, random forest analyses revealed a complementary pattern: in the text condition, individual differences in these same neural indices showed higher predictive importance, indicating that variability in neural efficiency became a stronger determinant of performance under elevated cognitive load.

This pattern reflects two distinct aspects of neural function. First, the color format was associated with descriptively higher average neural efficiency across all children, consistent with reduced cognitive demands. Second, the text format was associated with a greater role of individual neural capacity. When semantic interference increased, the children’s ability to maintain efficient prefrontal processing became more critical for successful performance. This aligns with cognitive load theory, specifically the reduction in extraneous load, while preserving intrinsic task demands, which posits that when extraneous demands are high, individual capacity for managing cognitive resources becomes a limiting factor [24]. Prior evidence supports this interpretation, showing that elevated cognitive load disrupts inhibitory processing and exacerbates individual differences in performance [68].

The relatively modest cross-validated *R*^2^ values (modest cross-validated performance in residualized models) reflect the inherent complexity of predicting individual performance from neural and psychometric measures alone. These models were designed to estimate the relative contribution of heterogeneous features rather than to achieve precise behavioral forecasting [69]. From this perspective, the key insight is not the absolute predictive accuracy but rather the shift in variable importance: neural indices became dominant predictors once age and general cognitive ability were controlled, accounting for 35% to 44% of residual variance. This underscores that PFC network efficiency captures performance variance distinct from broader developmental and intellectual factors.

Taken together, these findings have direct implications for the design of digital assessment tools for children. For clinical diagnostic guidelines, age-based normative standards should serve as a primary framework, given the dominant role of age in predicting performance. However, age-based norms and interface optimization are complementary: although norms ensure appropriate score interpretation across developmental stages, optimized response formats can reduce construct-irrelevant variance and improve measurement precision at every age level. The observation that the interaction format influences behavioral performance and clinical validity, with suggestive trends in neural efficiency, underscores the need to explicitly consider cognitive load when designing Stroop-based assessments. Specifically, reducing extraneous semantic demands through color-based response formats may enhance both measurement accuracy and ecological validity in pediatric populations.

### Limitations

This study has several limitations. First, although the within-subject design controlled for stable individual differences, the fixed task order (color followed by text) introduces the possibility of practice or fatigue effects. However, practice effects would be expected to benefit the second condition, potentially reducing rather than inflating the observed differences. The fact that the color condition showed superior performance despite being administered first suggests that the format effects were robust to any potential order confounds. Second, the sample comprised typically developing children aged 6-12 years, limiting the generalizability to broader developmental stages and clinical populations. Third, the neural feature set was necessarily coarse, relying on global efficiency and condition-level ∆FC; finer-grained metrics, such as event-locked or channel-specific analyses, may offer greater sensitivity. Finally, inferences about feature contributions relied on random forest models with modest predictive performance (*R*^2^=9%-23%). These results should be regarded as exploratory estimates of relative importance rather than robust predictive models. Nonetheless, they provide a valuable starting point for understanding the complex interplay between interface design and neurocognitive function in children.

### Future Directions

Future work should (1) counterbalance the task order and include repeated sessions to assess reliability; (2) extend the age range and incorporate clinical subgroups to evaluate external validity; (3) use more sensitive neural measures, including time-resolved connectivity and channel-specific analyses; and (4) apply mediation or structural equation models in preregistered analyses to formally test whether neural indices statistically account for format-related performance differences.

### Conclusion

The findings of this study demonstrate that the interaction format influences both cognitive load and measurement validity in tablet-based Stroop assessments for children. Color-based response options, which reduce extraneous semantic processing demands, yield better behavioral performance and stronger correlations with clinical attention measures compared to text-based options. Random forest analyses further revealed that neural indices of prefrontal efficiency become the dominant predictors of individual differences once age and general cognitive ability are controlled.

These results underscore the importance of interaction format calibration in pediatric digital assessments. To the best of our knowledge, this study provides some of the first empirical evidence directly comparing how the response format affects both neural indices of cognitive load and clinical validity in a pediatric sample, extending cognitive load theory from educational settings into digital health assessment design. As digital cognitive assessments become more prevalent in research and clinical settings, careful attention should be paid to how interface design shapes cognitive load for ensuring that these tools are both developmentally appropriate and clinically valid. For Stroop-based attention assessments in children, this work offers initial guidance: prioritizing response formats that minimize extraneous cognitive demands may enhance both measurement fidelity and clinical utility.

## Abbreviations

BASC-2: Behavior Assessment System for Children, Second Edition
FC: functional connectivity
fNIRS: functional near-infrared spectroscopy
FRI: Fluid Reasoning Index
FSIQ: full-scale intelligence quotient
IQ: intelligence quotient
K-WISC-V: Korean version of the Wechsler Intelligence Scale for Children, Fifth Edition
PFC: prefrontal cortex
PSI: Processing Speed Index
SNR: signal-to-noise ratio
VCI: Verbal Comprehension Index
VSI: Visual Spatial Index
WMI: Working Memory Index

## Data Availability

The datasets generated and analyzed during this study are available from the corresponding author upon reasonable request.
